# Levosimendan in Cardiac Arrest (LeiCA): Protocol for a Randomized, Double‐Blind, Phase II Clinical Trial

**DOI:** 10.1111/aas.70159

**Published:** 2025-11-24

**Authors:** Susanne Rysz, Benjamin Flam, Magnus Hedberg, Karolina Galmén, Johan Mårtensson, Therese Djärv, Malin Jonsson Fagerlund

**Affiliations:** ^1^ Perioperative Medicine and Intensive Care, Karolinska University Hospital Stockholm Sweden; ^2^ Section of Anesthesiology and Intensive Care Medicine, Department of Physiology and Pharmacology Karolinska Institutet Stockholm Sweden; ^3^ Rapid Response Car, Ambulance Care in Greater Stockholm Ltd. (AISAB) Stockholm Sweden; ^4^ Department of Anesthesiology and Intensive Care Danderyd Hospital Stockholm Sweden; ^5^ Department of Clinical Sciences Danderyd Hospital, Karolinska Institutet Stockholm Sweden; ^6^ Ambulance Helicopter, Ambulance Care in Greater Stockholm Ltd. (AISAB) Stockholm Sweden; ^7^ Medical Unit Acute/Emergency Department Karolinska University Hospital Stockholm Sweden; ^8^ Division of Clinical Medicine, Department of Medicine Solna Karolinska Institutet Stockholm Sweden

## Abstract

**Background:**

Despite developments in advanced life support, outcomes after out‐of‐hospital cardiac arrest (OHCA) remain poor. Levosimendan, a calcium‐sensitizing inodilator commonly used in heart failure, has shown beneficial effects in several experimental models and anecdotal clinical cases of cardiac arrest. The Levosimendan in Cardiac Arrest (LeiCA) pilot trial will evaluate levosimendan as an adjunct to advanced life support in refractory OHCA.

**Methods:**

This will be an investigator‐initiated, randomized, double‐blind, placebo‐controlled, single‐center pilot trial. Patients aged 18–75 years with witnessed, nontraumatic, refractory OHCA in Stockholm County, Sweden, will be eligible for enrollment by a physician‐staffed emergency medical service unit. Forty patients will be randomized in a 3:1 ratio to receive a single intravenous bolus of either levosimendan 2.5 mg (*n* = 30) or placebo (*n* = 10), administered during cardiopulmonary resuscitation via prefilled, masked syringes. Both groups will receive routine advanced life support and postresuscitation care as applicable. Included patients eligible for hospital transfer will be admitted to Karolinska University Hospital in Solna, Stockholm, Sweden. The primary endpoint is 30‐day survival. Secondary endpoints include additional clinical outcome measures.

**Conclusion:**

The LeiCA pilot trial will investigate the effects of levosimendan as an adjunct to advanced life support for refractory OHCA. The results will inform the design of a definitive multicenter trial.

**Trial Registration:**

ISRCTN15862521; October 22, 2025

## 1 Introduction

Out‐of‐hospital cardiac arrest (OHCA) affects approximately 6000 individuals annually in Sweden, yet overall 30‐day survival remains below 14% [1]. Beyond early cardiopulmonary resuscitation (CPR) with chest compressions and defibrillation, few pharmacological interventions have demonstrated improved long‐term outcomes [2]. Epinephrine, and amiodarone for shockable rhythms, remain standard components of advanced life support [3], despite no clear evidence of improved neurological outcomes [4, 5]. Moreover, epinephrine's effect size on survival appears modest [6], and may be even smaller in patients with initial shockable compared to nonshockable rhythms [7]. Amiodarone may increase survival to hospital admission relative to placebo [8], although this finding has been challenged [5].

The pathophysiology of cardiac arrest is complex, involving global ischemia and subsequent reperfusion injury. Postcardiac arrest syndrome, characterized by myocardial dysfunction, brain injury, and systemic inflammation, is another major contributor to poor outcomes [9]. Contemporary evidence suggests that current pharmacotherapy using epinephrine may exacerbate these processes by impairing cerebral microcirculation and thus worsening outcome [10, 11].

Levosimendan is a calcium sensitizer and inodilator [12], with documented efficacy and safety in acute decompensation of chronic heart failure [13, 14]. Its applications have expanded to various other clinical conditions and settings [15, 16]. With regard to cardiac arrest, experimental data suggest that levosimendan may facilitate ROSC [17, 18, 19], improve postresuscitation cardiac function [20, 21, 22, 23, 24, 25, 26], and enhance survival [20, 25, 27, 28] and neurologic recovery [24, 29]. Clinical reports recount successful application in refractory cases [30, 31, 32], and for postresuscitation myocardial dysfunction [33, 34, 35]. Its favorable safety profile and potential to mitigate the postcardiac arrest syndrome make it a promising candidate for further investigation in OHCA.

Given the lack of recent advances in resuscitation pharmacology, and the promising preclinical data on levosimendan, we propose a pilot trial to explore the 30‐day survival after intra‐arrest administration of levosimendan as an adjunct to advanced life support in refractory OHCA. Secondary outcomes will include key clinical and functional measures. The results will inform the design of a larger, definitive trial.

## 2 Methods

### 2.1 Trial Design

**FIGURE 1:**
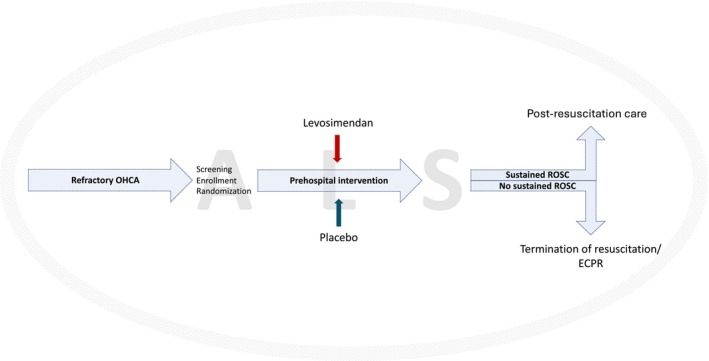
Study flow diagram. ALS, advanced life support; ECPR, extracorporeal cardiopulmonary resuscitation; OHCA, out‐of‐hospital cardiac arrest; ROSC, return of spontaneous circulation.

This will be an investigator‐initiated, randomized, double‐blind, placebo‐controlled, single‐center pilot trial conducted in Stockholm County, Sweden. It is designed to explore the effect of levosimendan, administered prehospitally during ongoing advanced life support, on 30‐day mortality. A schematic study flow diagram is presented in Figure 1. The estimated study period is 18 months. The study is preregistered in the Clinical Trials Information System (CTIS) (EU Clinical Trial No.: 2024‐517279‐20‐02) and ISRCTN at https://www.isrctn.com/ (ISRCTN15862521; October 22, 2025). The results will be reported according to the CONSORT Statement [36].

### 2.2 Inclusion Criteria

To be eligible for inclusion in the trial, patients must meet all of the following criteria:

1. Age 18–75 years
2. Witnessed OHCA
3. Prompt start of CPR (i.e., within 2 min)
4. First recorded rhythm ventricular fibrillation or pulseless ventricular tachycardia, or pulseless electrical activity in cases of suspected pulmonary embolism (as determined by the attending emergency medical service [EMS] physician)
5. Refractory cardiac arrest, that is, sustained beyond third rhythm check
6. Intravenous access

### 2.3 Exclusion Criteria

Patients must not be included in this trial if *any* of the following criteria are met:

1. Traumatic cause of cardiac arrest
2. First recorded rhythm asystole
3. Time to administration of study drug > 30 min after onset of cardiac arrest
4. Known or apparent pregnancy
5. Known preexisting advanced malignancy, severe neurological or systemic disease, advanced cardiac or pulmonary disease, end‐stage chronic kidney disease requiring dialysis, or ongoing bleeding
6. Known preexisting (i.e., current) *Do Not Attempt CPR* order

### 2.4 Screening and Randomization

Screening and enrollment will be conducted by the attending EMS physician, who assesses whether resuscitation attempts are appropriate and whether the patient meets all eligibility criteria. Eligible patients will then be enrolled and randomized in a 3:1 ratio (levosimendan:placebo) using block randomization with a block size of four.

Given the emergency setting, prefilled, numbered syringes will be supplied to each EMS team. These visually identical syringes contain either levosimendan or placebo, thereby ensuring blinding of patients, clinicians, and trial personnel. Allocation is operationalized by administration of the study drug. Patients will be enrolled consecutively.

### 2.5 Intervention

Patients randomized to the treatment group will receive a single intravenous bolus of levosimendan (2.5 mg in 49 mL of 5% glucose; concentration 0.05 mg/mL) administered during CPR via a prefilled, blinded syringe. Patients in the placebo group will receive an equivalent intravenous injection of 50 mL of 5% glucose, also administered during CPR from a prefilled, blinded syringe. The study drug will be administered manually as rapidly as possible and only once. Included patients will be transferred to Karolinska University Hospital in Solna, Stockholm, Sweden, unless resuscitation efforts are terminated out‐of‐hospital.

### 2.6 Blinding

The study drug will be prepared in‐hospital by personnel not involved in the trial, and dispensed into identical, blinded syringes bearing neutral study labels. This ensures blinding of patients, EMS physicians, paramedics, and the trial team. Only an external research nurse, independent of the study, will have access to the randomization list linking syringe numbers to treatment allocation.

### 2.7 General Management of Advanced Life Support and Postresuscitation Care

General management of cardiac arrest will follow local protocols both in the out‐of‐hospital and in‐hospital settings. Adherence to current European and national guidelines for advanced life support and postresuscitation care is strongly recommended. The use of levosimendan at established doses as an adjunct to intensive care after hospital admission will remain at the discretion of the treating physician.

### 2.8 Study Outcomes

The primary endpoint is 30‐day survival.

Secondary endpoints comprise a series of clinical outcomes intended to capture both early and downstream effects of the intervention. These include:

- Conversion to a potentially perfusing rhythm (except ventricular tachycardia) among participants without such rhythm at study inclusion;
- Return of spontaneous circulation (any, or sustained [≥ 20 min]), defined as the return of spontaneous pulse or blood pressure as determined by the treating clinician;
- Survived event, defined as ROSC sustained until arrival at the emergency department and transfer of care to medical staff at the receiving hospital;
- Transport to hospital;
- Hospital arrival status (i.e., condition at hospital arrival: ROSC, CPR in progress, deceased);
- Number and duration of vasopressor infusions (duration > 1 h) in the intensive care unit (ICU) up to 72 h (e.g., norepinephrine, epinephrine, phenylephrine, vasopressin, dopamine, methylene blue);
- Number and duration of inotrope infusions (duration > 1 h) in the ICU up to 72 h (e.g., epinephrine, dobutamine, milrinone, levosimendan);
- Mechanical circulatory support in the ICU within 7 days (e.g., veno‐arterial extracorporeal membrane oxygenation, Impella device, intra‐aortic balloon pump);
- Type and degree of organ dysfunction (e.g., renal, hepatic, or cardiac; exploratory);
- Neurological outcome at hospital discharge (i.e., cerebral performance category [CPC] 1–2 vs. 3–5);
- Circumstances of death;
- Plasma concentration of levosimendan and its metabolite (repeated samplings during the first 72 h); and
- Organ donation (i.e., ≥ 1 solid organ donated for transplantation).

### 2.9 Adverse Events

Patients experiencing cardiac arrest commonly develop complications related to the precipitating cause, the acute condition itself and associated global ischemia–reperfusion injury, resuscitation efforts (e.g., chest compressions, defibrillation), and postresuscitation care. Such complications may include circulatory instability, arrhythmias, organ dysfunction or failure, and metabolic and electrolyte disturbances. Thus, distinguishing adverse events directly attributable to the intervention from those inherent to the underlying condition is challenging.

In this trial, potential adverse events will be systematically recorded and compared between treatment groups to identify any imbalance suggestive of a drug‐related effect. Clinical data will be collected up to 3 days following inclusion, and medical records will be reviewed at Day 30 to capture any delayed or secondary effects. At Day 30, medical records will be scanned for other potential side effects. Particular attention will be paid to hemodynamic instability, including recurrent or prolonged hypotension, increased requirements for vasoactive agents or mechanical circulatory support, and new or worsening arrhythmias. Only events that are unexpected or considered possibly related to the study drug will be reported to and reviewed by the trial management committee.

### 2.10 Follow‐Up

Follow‐up will be conducted using prospectively collected clinical data. Information on 30‐day survival, neurological outcome (assessed using the CPC scale), and organ function will be obtained from electronic medical records. No additional in‐person visits or direct contact with participants or their next of kin will be required.

### 2.11 Data Collection and Management

Individual participant data, including background characteristics, clinical findings, laboratory results, and resuscitation metrics such as CPR quality and timing, will be collected from the EMS physician, ambulance reports, and medical records. Key variables will include neurological status, hemodynamic parameters, organ function and medication use (e.g., vasoactive and inotropic agents), and the need for mechanical circulatory support.

All data will be entered by site personnel into a secure, web‐based electronic case report form. The trial database will be hosted and maintained on a secure server managed by the study center.

### 2.12 Sample Size and Power Estimations

As this is, to the best of our knowledge, the first trial designed to investigate the effect of intra‐arrest levosimendan in OHCA, no prior effect estimates are available. The purpose of this pilot study is to explore the effects of levosimendan compared with placebo and to estimate the potential effect size on 30‐day survival and secondary outcomes. Accordingly, no formal power calculation was performed; we estimated that enrolling 40 patients would be sufficient to detect a potential signal of effect.

### 2.13 Statistical Analyses

All analyses will follow the intention‐to‐treat principle, with complementary per‐protocol analyses performed to assess drug‐specific effects. Continuous variables will be summarized as mean ± standard deviation or with 95% confidence intervals, or as median with interquartile range, as appropriate. Categorical variables will be reported as counts and percentages.

The primary outcome will be analyzed using Cox proportional hazards regression and illustrated with Kaplan–Meier survival curves. Sensitivity analyses of the primary outcome will include adjustment for baseline characteristics. Secondary outcomes will be compared between groups when applicable using appropriate statistical tests depending on the variable. All statistical tests will be two‐sided, with a significance level set at *p* < 0.05.

### 2.14 Ethics and Informed Consent

This study qualifies as emergency research, as participants are unconscious at the time of enrollment and unable to provide informed consent. Given the urgent nature of the intervention, which must be administered during advanced life support for OHCA, a delayed consent process will be employed. Written informed consent will be sought from surviving patients as soon as they regain the capacity to make an informed decision. In the interim, the patients' legal representatives will be informed of the study participation as soon as practically possible. Ethics approval permits the use of data from deceased participants without consent to minimize survivor bias.

The trial will be conducted in accordance with this clinical trial protocol, the EU Clinical Trials Regulation (EU No. 536/2014), the Declaration of Helsinki, the International Council for Harmonisation guideline for Good Clinical Practice (ICH‐GCP), and all applicable national regulations. The study has been submitted to CTIS (EU Clinical Trial No.: 2024‐517279‐20‐02) and was approved by the Swedish Ethical Review Authority and the Swedish Medical Products Agency (No.: 5.1.1‐2025‐025629; June 11, 2025).

### 2.15 Data Safety Monitoring and Interim Analysis

The trial will be monitored by an independent monitor before, during, and after completion. Monitoring ensures compliance with the protocol, ICH‐GCP, and regulatory requirements, and that patient rights, safety, and data integrity are maintained.

### 2.16 Trial Status and Timeline

This pilot trial is scheduled to start in January 2026 and is estimated to run for 18 months.

## 3 Discussion

Despite advances in resuscitation and intensive care, only a minority of patients who suffer OHCA experience a favorable outcome. The lack of new effective treatment options highlights a critical gap in current care and underscores the urgent need for further research. In this context, levosimendan emerges as a promising candidate for evaluation. It has demonstrated beneficial effects in multiple preclinical cardiac arrest models [17, 18, 19, 20, 21, 22, 23, 24, 25, 26, 27, 28, 29]. Additionally, several case reports have described successful resuscitation in cardiac arrest patients treated with levosimendan under compassionate use [31, 32]. Levosimendan's robust safety profile, established in cardiac and intensive care populations [13, 14, 16], further supports its potential suitability for use in the vulnerable cardiac arrest population.

The study population is restricted to patients aged 18–75 years without advanced comorbidities, presenting with witnessed, nontraumatic, primarily shockable, refractory OHCA. This approach ensures a more homogeneous cohort with a reasonable likelihood of survival and measurable treatment effect. The selection further aims to reduce heterogeneity in etiology and time to intervention, facilitating interpretation of the results. From a practical standpoint, the eligibility criteria largely mirror those of the regional extracorporeal CPR program [37], supporting efficient screening and recruitment.

In the current study protocol, we opted for intravenous bolus administration of levosimendan at a fixed dose of 2.5 mg, corresponding to approximately 31 μg/kg for an 80 kg individual. Given the time‐critical and complex nature of advanced life support for OHCA, we selected a simplified, one‐size‐fits‐all dosing strategy with a rapid and efficient mode of administration, analogous to established pharmacological adjuncts (i.e., epinephrine and amiodarone). In the absence of recognized dosing guidelines for this indication, dose selection was guided by available preclinical, clinical, and pharmacological data. Premarketing studies demonstrated that doses up to 10 mg of racemic simendan (equivalent to 5 mg of levosimendan) were well‐tolerated in healthy volunteers [38], with the most favorable hemodynamic profile observed after 2 mg of levosimendan [39]. A suggested loading dose of levosimendan for patients with decompensated heart failure is 6–12 μg/kg over 10 min [40]. Clinical case reports of levosimendan use in refractory cardiac arrest describe diverse administration strategies, typically including a rescue bolus of 10–35 μg/kg [31, 32]. A recent placebo‐controlled large‐animal cardiac arrest study from our group, using human equivalent doses of 11 μg/kg (low‐dose) and 59 μg/kg (high‐dose), demonstrated a dose‐dependent improvement in hemodynamics [19].

Beyond evaluating a novel application for levosimendan, the proposed pilot trial is pioneering in another aspect. Unconscious patients, often the most critically ill, have historically been excluded from clinical drug trials in Sweden due to strict informed consent regulations [41]. Consequently, this high‐risk population has been underrepresented in research, limiting progress in developing therapies that could meaningfully improve survival and recovery. To the best of our knowledge, this study is the first contemporary Swedish clinical drug trial in OHCA to obtain approval, marking an important step toward more inclusive and clinically relevant emergency research in Scandinavia.

### 3.1 Strengths and Limitations

A key strength of this pilot study is the robust preclinical evidence supporting levosimendan in cardiac arrest, demonstrating improved outcomes across several animal models. Since its market introduction in Sweden in 2000, levosimendan has become one of the most extensively studied cardiovascular drugs, with a substantial evidence base and broad clinical experience in severely ill patients with cardiac failure. Safety data are reassuring, with few major adverse events apart from hypotension reported when used as indicated [14]. Conducting the pilot trial at a single center with comprehensive resuscitation resources, around‐the‐clock interventional cardiology, extracorporeal CPR, cardiothoracic surgery and intensive care, and pharmacologic laboratory support, further strengthens feasibility and relevance. The pilot study design is essential for generating preliminary data on clinical outcomes, safety, and feasibility in this challenging setting.

Limitations include the small sample size and single‐center design, which constrain statistical power and limit generalizability.

## 4 Conclusion

The proposed pilot trial will investigate levosimendan as an adjunct to epinephrine and amiodarone during advanced life support in patients with OHCA. Building upon preclinical data in cardiac arrest and extensive clinical experience from other settings, the study aims to yield valuable insights into levosimendan's impact on clinical outcomes, as well as its safety and feasibility in the current setting. The findings may inform the design of future multicenter trials and contribute to the development of evidence‐based treatment strategies in one of the most critical areas of emergency care.

## Author Contributions

S. Rysz and B. Flam drafted the manuscript together with T. Djärv and M. Jonsson Fagerlund. All authors contributed to the study design and critically revised the manuscript.

## Funding

The study is supported by ALF project grants from Region Stockholm (FoUI‐541731 [S. Rysz] and FoUI‐973308 [M. Jonsson Fagerlund]), the David and Astrid Hagelén Foundation (S. Rysz), the Swedish Society of Medicine (S. Rysz), the Swedish Heart Lung Foundation (M. Jonsson Fagerlund), and the departments and institutions involved.

## Conflicts of Interest

The authors declare no conflicts of interest.

## Data Availability

Data sharing not applicable to this article as no datasets were generated or analysed during the current study.
